# Ground Tire Rubber in the Sustainable Development of Flexible and Conductive Thermoplastic Polyurethane/Carbon Black Composites

**DOI:** 10.3390/polym18060741

**Published:** 2026-03-18

**Authors:** Krzysztof Formela, Mateusz Cieślik

**Affiliations:** 1 Department of Polymer Technology, Faculty of Chemistry, Gdańsk University of Technology, Gabriela Narutowicza 11/12, 80-233 Gdańsk, Poland; 2 Advanced Materials Center, Gdańsk University of Technology, Gabriela Narutowicza 11/12, 80-233 Gdańsk, Poland; mateusz.cieslik@pg.edu.pl; 3 Institute of Nanotechnology and Materials Engineering, Faculty of Applied Physics and Mathematics, Gdańsk University of Technology, Gabriela Narutowicza 11/12, 80-233 Gdańsk, Poland

**Keywords:** ground tire rubber, conductive polymer composites, structure-property relationships

## Abstract

Ground tire rubber (GTR) is composed of high-quality components; therefore, searching for new technologies for GTR recycling and upcycling is fully justified. In this work, the effect of micronized ground tire rubber content on the rheological, mechanical, thermal, and morphological properties, electrical conductivity, and electrochemical behavior of thermoplastic polyurethane/carbon black was investigated. The application of micronized ground tire rubber in the range of 5–20 wt% reduces the manufacturing cost by 5.6–22.6% and improves the electrical conductivity and electrochemical properties of composites. The results showed that higher contents of ground tire rubber increased the electrical conductivity of the studied materials from 11.7 to 33.8 S/m. This phenomenon is due to two factors: (i) additional carbon black present in GTR and (ii) phase separation that promotes local carbon-rich domains and facilitates conductive pathway formation. Electrochemical analysis revealed that the studied composites after laser activation can be used as flexible sensors. This research work confirms that using a ground tire rubber as a low-cost and valuable source of raw materials is a promising approach for the sustainable development of soft electronics.

## 1. Introduction

Thermoplastic elastomers (TPE) are an interesting group of polymers, which, according to ASTM D 883 (“Standard Terminology Relating to Plastics”), are a “diverse family of rubberlike materials that, unlike conventional vulcanized rubbers, can be processed and recycled like thermoplastic materials”. Unique processing and performance properties, combined with the ease of recycling of thermoplastic elastomers led to their widespread use in various industries, such as automotive, construction and building materials, sports goods and toy applications, etc.

One of the promising routes for the new applications of thermoplastic elastomers is the development of high-performance soft electronics [1], such as stretchable and wearable sensors [2] or highly flexible electromagnetic interference shields [3].

The Smithers report indicated that, in 2021, the thermoplastic elastomer market was around 3.84 million tonnes and was expected to grow to 5.55 million tonnes by 2026 [4]. This confirms the growing demand for the further development and application of this group of materials, especially in the automotive industry, which is estimated to reach 2.46 million tonnes in 2026 (around 44.3% of the global consumption market of TPE).

Generally, thermoplastic elastomers are produced through two methods: polymerization [5] and the melt-compounding of semi-crystalline plastomers and amorphous elastomers [6]. The possibility of manufacturing new thermoplastic elastomers with simple mixing of commercial polymers and additives using equipment commonly used in the industry, such as internal mixers and extruders, offers a huge advantage. This approach allows us to tailor the processing and performance properties of thermoplastic elastomers through the proper selection of the thermoplastic/elastomer blend composition and/or optimization of their processing method or conditions [7,8,9].

The mixing of plastomers and rubbers reduces the costs related to the development and further upscaling of new thermoplastic elastomers because it eliminates the complicated steps and protocols necessary for a controlled polymerization process. This strategy of TPE manufacturing is available for more companies and increases competitiveness, which might have a positive impact on innovations and higher chances of discovering new applications. Considering environmental and economic aspects, the development of thermoplastic/elastomer blends based on waste plastics and/or rubbers is fully justified [10,11,12]. Ground tire rubber (GTR) is composed mainly of high-quality rubbers and carbon black [13], which should be considered as low-cost and valuable sources of raw materials.

Many studies have been carried out on the development of thermoplastic/GTR systems, which were comprehensively described in works [14,15,16]. Generally, a higher content of GTR in thermoplastic/GTR systems caused difficulties with their processing and resulted in the deterioration of their mechanical or thermal properties, which is due to the cross-linked nature of GTR and limited interfacial adhesion between the GTR particles and matrix. Therefore, most research works are aimed at the assessment of the processing, microstructure, and mechanical and thermal properties of thermoplastics/GTR systems as a function of the used compatibilization strategy and/or processing conditions.

Recently, more and more attention is focused on the specific applications of ground tire rubber, such as dedicated composites for additive manufacturing technologies [17,18] or segregated structures for electromagnetic interference shielding [19,20,21]. This approach is interesting due to two main reasons. First, there are obvious environmental and economic aspects related to the application of GTR. Second, the low compatibility of cross-linked rubber particles with other polymer matrices should be considered as an advantage for segregated structure formulation.

However, so far, only a few research groups have explored this opportunity for conductive polymer composite development. These studies focused on conductive polymer composites modified with cross-linked rubber particles and carbon nanotubes [22,23,24]. The results obtained by independent research groups clearly show that cross-linked rubber particles in polymer/carbon nanotube composites might promote carbon nanotube domains and facilitate conductive pathway formation. However, the studies on the effects of other commonly used conductive nanofillers (e.g., carbon black, graphite, graphene nanoplatelets) and GTR on the electrical properties of polymer composites are rather limited [25]. Therefore, further investigation of various polymer/GTR systems modified with conductive nanofillers is fully justified.

In this field of research, the development of high-performance and flexible thermoplastic polyurethanes/GTR composites seems to be a very promising approach, especially considering the commercial availability of thermoplastic polyurethanes, characterized by a wide range of performance properties and potential applications [26,27].

However, to the best of our knowledge, there is no published information about the role of GTR in the sustainable development of flexible and conductive thermoplastic polyurethane/carbon black composites dedicated for soft electronics with a special attention on the electrical conductivity or electrochemical properties.

In this work, flexible and electrically conductive polymer composites were prepared from thermoplastic polyurethane powder filled with carbon black and ground tire rubber. The effect of the GTR content (in the range of 5–20 wt%) on the rheological, mechanical, thermal, and morphological properties, electrical conductivity and electrochemical behavior of thermoplastic polyurethane/carbon black composites was investigated.

## 2. Materials and Methods

### 2.1. Materials

Thermoplastic polyurethane (TPU) powder grade Flexa performance was from Sinterit Sp. z o.o. (Cracow, Poland). According to the technical data sheet, the TPU powder is a high-strength, flexible material with a mean particle size in the range of 70–90 μm and a melting temperature in the range of 120–150 °C.

Carbon black (CB) Ensaco 360G from Imerys Graphite & Carbon (Bironico, Switzerland) is characterized by a surface area of 780 m^2^/g and an oil absorption number of 320 mL/100 g. According to the literature, the mean particle size of carbon black Ensaco 360G is 30 nm [28].

MicroDyne 184-TR from Lehigh Technologies (Olsztyn, Poland) is micronized rubber powder with an average particle size of 0.162 mm manufactured from ground tire rubber (GTR) using cryogenic turbo mill technology. Micronized GTR was selected because the lower particle size of GTR usually reduces its negative effect on the tensile properties of polymer/GTR composites [29,30].

### 2.2. Sample Preparation

#### 2.2.1. High-Speed Mixing

Polyurethane/carbon black composites modified with micronized ground tire rubber particles were prepared through high-speed mixing using an analytical mill model A11 from IKA-Werke GmbH & Co. KG (Staufen im Breisgau, Germany). All components were dried at 60 °C before use. Composite materials were mixed with a rotor speed of 25,000 rpm for 30 s. The total weight of components used for composite preparation through high-speed mixing was 10 g per batch. Sample composition and coding are summarized in Table 1.

All tested composites contain the same amount of conductive carbon black (15 wt%) to reach an acceptable electrical conductivity of the studied material. Micronized GTR was used at 5–20 wt% as a low-cost substitute of TPU powder, and the prepared composites were coded as TPU/CB/GTRX, where X denotes the content of GTR used in wt%. For example, sample coded as TPU/CB/GTR5 is composed of 80 wt% of TPU, 15wt% of CB and 5 wt% of GTR.

#### 2.2.2. Compression Molding

TPU/CB/GTR composites were formed at 200 °C and at a compression of 100 bars into 0.6 mm thick tiles, 100 × 120 mm, using a PH-90 hydraulic press manufactured by ZUP Nysa (Nysa, Poland). During sample preparation, PTFE-coated fiberglass fabric was used to prevent the adhesion of TPU/CB/GTR composites to steel form. The total time necessary for composite formulation was 10 min. In the first step, composite material in the form of powder was preheated at 200 °C for 4 min; then, the preheated sample was compression molded for 1 min at 200 °C under a pressure of 100 bar. After compression, the sample was cooled for 5 min at 100 bar.

#### 2.2.3. Laser Activation

Sample surface activation for electrochemical measurements was performed using a LaserTEC ECO 320 CO_2_ from Tech-CNC (Ziębice, Poland) with a maximum laser power (LP) of 40 W. Surface activation was achieved at 10% and 20% of the maximum laser power, respectively.

### 2.3. Methodology

The increment of temperature after high-speed mixing (ΔT_mixing_), defined as the difference between the temperature of a material before and after mixing, was measured using an infrared thermometer Fluke 62 MAX+ from Fluke Corporation (Everett, WA, USA).

The processing and flowability of prepared materials were investigated using melt mass–flow rate (MFR) and melt volume–flow rate (MVR) at 190 °C according to ISO 1133 using an mFlow plastometer from ZwickRoell (Ulm, Germany). Depending on the flow characteristics of the samples, loads of 10 kg and 15 kg were used. The melt density (d_m_) at 190 °C was calculated using the following Equation (1):

(1) $$d_{m}=\frac{MFR}{MVR}$$

Electrical properties were studied directly on the samples prepared through compression molding or on the samples collected after MFR measurements. The distance between measurement points was 10 cm. The resistance (R) of the samples was measured with a UNI-T UT139C digital multimeter from Uni-Trend Technology Co., Ltd. (Dongguan, China), while resistivity (ρ) was calculated using the following Equation (2):

(2) $$\rho =R\times \frac{A}{l}$$

where R is resistance (Ω), A is the cross-sectional area of the sample (m^2^), and l is the length of the sample (m).

Conductivity (σ) is the inverse of resistivity, as presented in Equation (3):

(3) $$\sigma =\frac{1}{\rho }$$

Differential scanning calorimetry (DSC) was performed on a DSC 204 F1 Phoenix apparatus from Netzsch Group (Selb, Germany). DSC analysis was conducted under a nitrogen atmosphere in the temperature range from −80 °C to 250 °C at a heating rate of 10 °C/min. First, samples were heated from 30 to 250 °C, then cooled down to −80 °C and heated again to 250 °C.

Thermal stability was investigated on 10 mg samples using a TG 209 apparatus from Netzsch Group (Selb, Germany). The test was carried out under nitrogen atmosphere in the temperature range of 35–800 °C, with a heating rate of 10 °C/min.

The tensile strength and elongation at break were measured based on ISO 37. Tensile tests were carried out on an AGS-10kNX machine from Shimadzu (Kyoto, Japan) at a constant speed of 500 mm/min using a dumbbell sample type 2 with thickness reduced to 0.6 mm. The reported results are an average of five measurements for each sample.

The hardness of studied materials on the Shore A scale was determined in accordance with ISO 48-4 using a Bareiss HPE III durometer (Oberdischingen, Germany). The reported results are the average of ten measurements per sample.

The density of the samples was measured using the Archimedes method in accordance with ISO 1183. Measurements were carried out at room temperature in methanol. At least three measurements were performed per sample.

The morphology of the surfaces created by breaking the samples in the tensile test at the speed of 500 mm/min was evaluated using a FlexSEM 1000 II scanning electron microscope from Hitachi Ltd. (Tokyo, Japan). During the analysis, the electron beam accelerating voltage was 15 kV. Before SEM analysis, the samples were coated with a thin gold layer in the vacuum chamber using a Cressington 108 Auto Sputter Coater (Watford, UK).

Fourier transform infrared spectroscopy (FTIR) analysis of the obtained materials was performed using an IRTracer 100 from Shimadzu (Kyoto, Japan). Analysis was performed in attenuated total reflectance mode using a germanium crystal at a 4 cm^−1^ resolution and 64 scans in the range 4000–500 cm^−1^.

The morphology of the samples after laser treatment was investigated using a digital microscope VHX-7000 from Keyence International (Mechelen, Belgium). The morphology of the samples was studied without further preparation before analysis.

Raman spectroscopy was performed using a Horiba Scientific XploRA Plus (Kioto, Japan) spectrometer. A 532 nm laser was used; the spectral acquisition times were 10 scans, with 15 s/scan. The spectra were collected within the region from 200 to 3400 cm^−1^.

Contact angle measurements were performed at room temperature on 2 µL deionized water drops using an Ossila goniometer (Sheffield, UK) with ±1° accuracy. Angle measurements were performed using ImageJ software ver. 1.54g and the Snake Drop algorithm.

All electrochemical measurements were performed with a Biologic-VSP300 potentiostat/galvanostat (Toulouse, France). The measurements were performed in a cell consisting of the three-electrode system: for TPU/CB, TPU/CB/GTR was the working electrode (diameter of 8 mm, measurement area of 0.564 cm^2^), Ag/AgCl (0.1 M NaCl) was the reference electrode, and platinum wire was a counter electrode. A 0.01 M phosphate buffer (pH 7.4) containing 2.5 mM K_3_[Fe(CN)_6_] was used as an electrolyte solution. Cyclic voltammetry (CV) was performed in the range from −0.4 V to 0.6 V at a scan rate of 100 mV/s. Differential pulse voltammetry (DPV) was obtained in the potential range from 0.1 V to +0.9 V, applied pulse amplitude 50 mV, potential step 10 mV, modulation time 0.07 s, and interval time 1.85 s. The limit of detection (LOD) and limit of quantification (LOQ) for the linear detection range of 0.25–5 mM of paracetamol were estimated using Equations (4) and (5):

(4) $$LOD=3.3\;\times \frac{RSD}{slope}$$

(5) $$LOQ=10\times \frac{RSD}{slope}$$

where RSD is the relative standard deviation in the low concentration range.

## 3. Results and Discussion

### 3.1. Cost Savings and Processing Behavior

**Figure 1:**
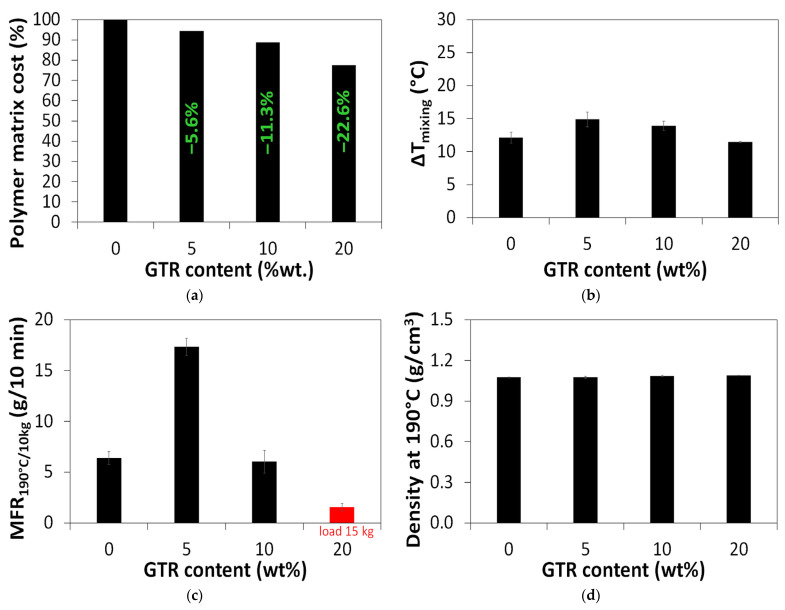
The effect of GTR content on TPU/CB properties: (**a**) cost savings; (**b**) increment of temperature after high-speed mixing; (**c**) MFR; (**d**) density at 190 °C.

The effect of GTR content on the cost savings related to TPU/CB composite preparation is presented in Figure 1a. Considering the current prices of polyurethane powder and micronized GTR with 5–20 wt% rubber recycling products in the studied materials, the resulting cost savings were 5.6–22.6%.

The increment of temperature before and after high-speed mixing (ΔT_mixing_) was measured using an infrared thermometer, and the results are presented in Figure 1b. It was found that high-speed mixing increased the temperature of the material in the range of 11.5–14.9 °C. This increase was slightly higher for samples modified with 5 wt% and 10 wt% GTR. Shear forces increase the friction between GTR particles and enhance exothermic reactions, leading to the self-heating of the cross-linked elastomer during thermo-mechanical treatment [31]. This phenomenon is due to the oxidation and devulcanization of GTR particles. For a sample with 20 wt% GTR, this effect seems to be negligible due to the limited interactions with other components and a higher level of voids in the composite material.

The processing and flowability of the studied materials were studied based on the MFR parameter, and the obtained results are presented in Figure 1c. This standardized parameter is very important in the development of materials dedicated to additive manufacturing technologies, especially fused deposition modeling technology.

Wang et al. [32] indicated that a threshold value of 10 g/10 min and a load of 2.16 kg (determined in accordance with ISO 1133) are put forward for a successful printing for temperature in the range of 190–220 °C, enabling the fast and practical screening of PLA-based filaments for 3D-printed materials. It seems that a similar level of threshold (determined in different loads and/or temperatures) can also be assumed for other materials dedicated to 3D printing.

Paszkiewicz et al. [33] showed that polypropylene modified with 10 wt% and 30 wt% GTR can be successfully used for filament production and 3D printing. The studied materials were characterized through MFR, determined at 230 °C and 2.16 kg, in the range of 6–8 g/10 min with ±25% tolerance.

As presented in Figure 1c, a higher content of GTR resulted in a decreased MFR value for the studied composites. This is related to the limited flowability of the material from the application of cross-linked rubber particles [34].

However, it was surprising that, for the TPU/CB/GTR5 sample, the MFR values were higher than in the case of the TPU/CB sample. This can be explained by the fact that, during high-shear mixing, main chain scission and cross-linking degradation affect the reduction in the composites’ viscosity [35]. This observation corresponds well with the highest ΔT_mixing_ measured for the TPU/CB/GTR5 sample (please see Figure 1b).

As presented in Figure 1d, the melt density at 190 °C determined for the studied materials was in the range of 1.07–1.09 g/cm^3^, and the effect of GTR content on the value of this parameter was negligible.

### 3.2. Resistance and Conductivity Assessment

**Figure 2:**
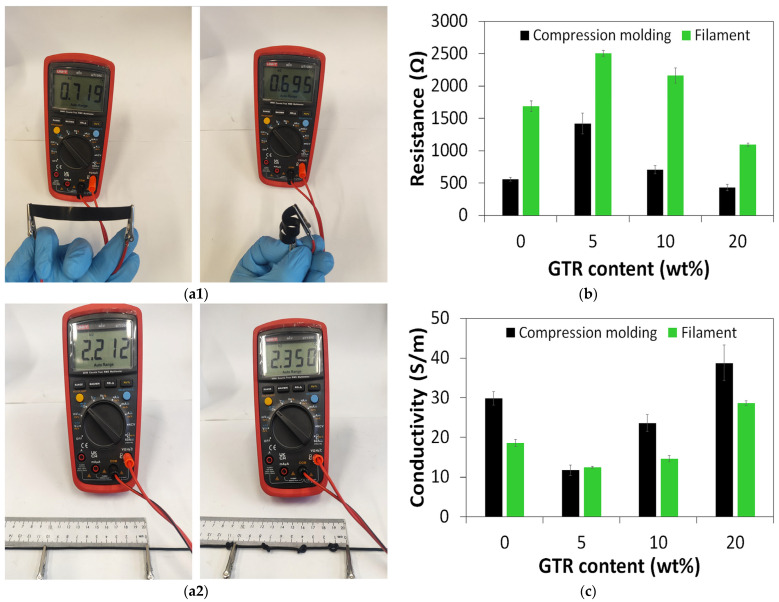
(**a1**,**a2**) The appearance of sample TPU/CB/GTR10 during resistance measurements; (**b**) resistance of studied materials measured by multimeter; (**c**) conductivity.

As presented in Figure 2(a1,a2), the measurement of the electrical properties of the studied material was performed using a simple and commonly available two-probe digital multimeter. This approach seems to be the best option for screening and preliminary investigation of electrical properties during the development of conductive polymer composites. This method is acceptable for comparative screening under identical measurement conditions, especially probe and sample dimensions, which affect contact resistance.

Two forms of materials were tested. The first form was rectangle samples (10 × 100 × 0.6 mm) cut directly from compression molding sheets. The second form of tested materials was filaments with an approximate diameter of 2 mm, formed during MFR measurements at 190 °C. For flexibility testing, the studied materials were rolled in the case of rectangle samples and tied in the case of filaments. This simple test is a good approach for a quick check of the flexibility of prepared materials and of their possible damage after the removal of external forces. All studied composites passed this simple test without damage.

The resistance and conductivity of the studied composites were determined on straight samples using two-probe measurements, with the length 100 mm, and the obtained results are presented in Figure 2b,c. As can be observed, the second processing of the studied composites through piston extrusion during the MFR test resulted in a significant increase in resistance and a decrease in conductivity. This is because the processing conditions of conductive polymer composites significantly affect the redistribution of disperse conductive filler particles and change in electrical conductivity profiles [36].

It was found that, regardless of sample preparation technique (directly from compression molding vs. samples after MFR test), the addition of micronized GTR up to 10 wt% to TPU/CB composites increased resistance and decreased conductivity compared to the reference sample. On the other hand, the TPU/CB/GTR20 sample with 20 wt% was characterized by better electrical properties than the TPU/CB sample, which is due to a higher content of carbon black in the system, with an increasing amount of GTR and a limited compatibility of GTR with the TPU matrix.

As can be observed, TPU/CB/GTR composites are characterized by a relatively high conductivity in the range of 11.7–33.8 S/m. For better analysis, the results obtained for TPU/CB/GTR were compared with the commercial conductive polymer composites reported in the literature.

Stankevich et al. [37] presented interesting research about the electrical resistivity of 3D-printed polymer elements based on two commercial conductive polymer composites: Proto-Pasta (polylactic acid/carbon black composite) and Koltron G1 (polyvinylidene fluoride/graphene composite). It was found that printing conditions and thermal postprocessing have a significant impact on the electrical conductivity of the studied materials, which was in the range of ~14–50* S/m (* values estimated from graphs).

Recently, Nowka et al. [38] performed comprehensive studies on the characterization of the electrical properties of 3D printing filaments and additively manufactured elements made from commercially available conductive polymer composites. The experimental results, using a four-point probe measurement technique, showed that the electrical conductivity of 3D printing conductive filaments ranged from 0.45 to 7692 S/m, with a median of 28.1 S/m.

This research work showed that the electrical conductivity of TPU/CB/GTR composites in the range of 11.7–33.8 S/m is very close to the median value reported for commercially available conductive polymer composites used in additive manufacturing.

### 3.3. Thermal Analysis

**Figure 3:**
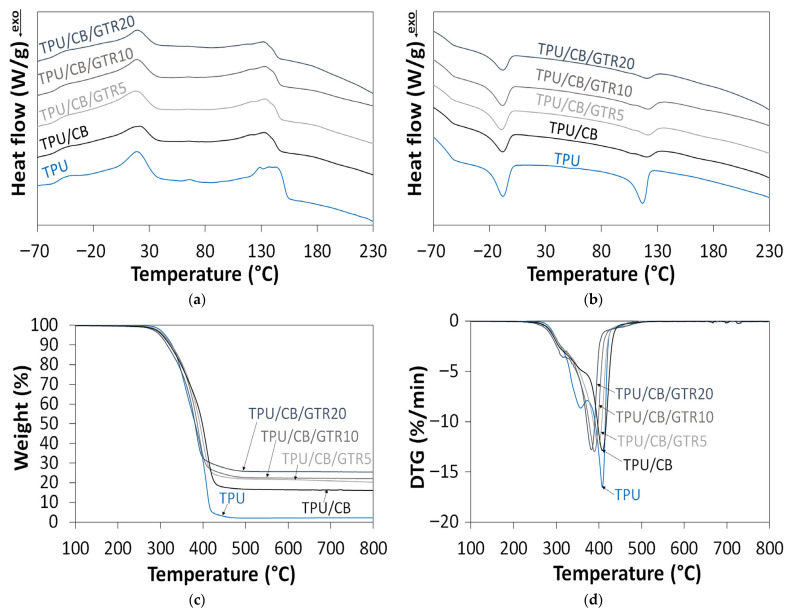
Thermal analysis of TPU/CB composites modified with GTR: (**a**) DSC curves: heating; (**b**) DSC curves: cooling; (**c**) TGA; (**d**) DTG.

Differential scanning calorimetry (DSC) was performed to investigate the effect of micronized GTR on the glass transition temperature, melting and crystallization behavior of the studied composites. The obtained results are presented in Figure 3a,b.

As can be noticed, the glass transition temperature of the studied materials slightly decreased with increasing GTR content, which can be attributed to two factors. The first is related to the glass transition temperature of GTR, which, according to the literature, is −61 °C [39]. The second factor is related to the limited adhesion between TPU and GTR, which might result in the increased material porosity with the higher GTR content and therefore the lower glass transition temperature.

Furthermore, it was found that TPU powder showed two melting and crystallization points. This indicates that TPU powder was modified with a flow and antistatistic agent, which reduces powder agglomeration and improves free-flowing characteristics. According to the literature, such kinds of additives are used at 0.05 to 1.5% by weight, depending on the application of the polymer powder [40].

For the studied materials, the first melting temperature (T_m1_) and crystallization temperature (T_c1_) were in the range of 18–22 °C (19 °C for pure TPU) and between −11 to −8 °C (−8 °C for pure TPU), respectively. Such low values of melting and crystallization temperatures suggest that the flow and antistatistic additive used was probably based on fatty acid derivatives [41]. The second melting (T_m2_) in the range of 132–133 °C (137 °C for pure TPU) and crystallization temperature (T_c2_) in the range of 121–122 °C (117 °C for pure TPU) are related to the polyurethane matrix and correspond well with the information provided by the producer (please see Section 2). It was observed that the application of carbon black to the TPU matrix reduced the melting temperature and increased the crystallization temperature compared to pure TPU. This is due to the nucleation effect of carbon black as a nanofiller. The enthalpies of melting and crystallization for the studied composites were significantly lower than those of pure TPU, which is attributed to the higher content of the amorphous phase of GTR.

The results showed that GTR had a negligible effect on the melting and crystallization temperatures of the studied materials. Some small changes in the thermal properties of the studied materials can be related to the presence of carbon black in GTR and its impact on the nucleation and crystallization process.

Thermogravimetric analysis (TGA) and derivative thermogravimetry (DTG) were used to investigate the effect of GTR content on the thermal stability of TPU/CB/GTR composites, and the obtained results are presented in Figure 3c,d and summarized in Table 2.

As can be observed, the GTR content significantly affects the thermal stability of TPU/CB/GTR composites. The application of carbon black and micronized GTR decreased the thermal stability of the studied materials compared to pure TPU. For example, T_−2%_, corresponding to the temperature related to a 2% weight loss, was 297 °C for pure TPU, 285 °C for TPU/CB and 279–290 °C for TPU/CB/GTR composites. The value of the T_−2%_ parameter decreased with an increasing content of GTR in TPU/CB/GTR composites. As could be expected, the char residue increases proportionally with the GTR content, from 16.0 wt% for TPU/CB to 25.6 wt% for TPU/CB/GTR20. This is due to the presence of carbon black and ash in GTR, with an average of 36.6 wt% [42].

The DTG curves of the studied materials show two maxima. In the case of TPU, the two maxima on the DTG curve correspond to hard segments T_max1_ ∼360 °C and soft segments T_max2_ ∼410 °C [43].

It was found that a higher GTR content decreased the T_max2_ value, which was in the range of 384–398 °C for TPU/CB/GTR composites. A similar finding was recently described by Zhou et al. [44], who studied composites based on devulcanized ground tire rubber and recycled polyurethane. The authors indicated that this phenomenon might be due to heterostructures formed by devulcanized ground tire rubber in the polyurethane matrix, which might have resulted in the non-uniform heating during the thermal decomposition process which, as a consequence, accelerated the material degradation kinetics.

### 3.4. Physico-Mechanical Properties

**Figure 4:**
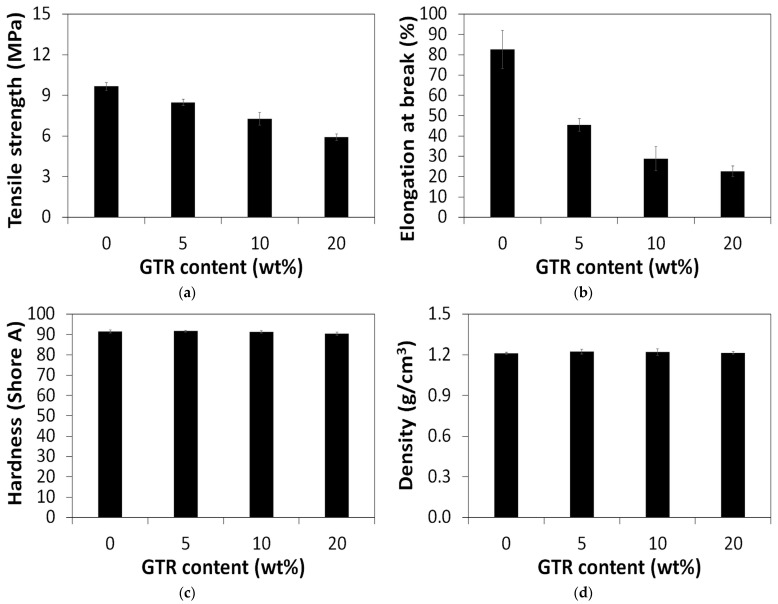
Physico-mechanical properties of studied composites: (**a**) tensile strength; (**b**) elongation at break; (**c**) hardness; (**d**) density.

The physico-mechanical properties of the studied composites are presented in Figure 4. It was observed that a higher content of GTR in TPU/CB/GTR resulted in a decrease in tensile strength from 9.7 MPa for the TPU/CB sample to 5.9 MPa for the TPU/CB/GTR20 sample (Figure 4a). A similar trend was observed for elongation at break, which decreased from 83% for the TPU/CB sample to 23% for the TPU/CB/GTR20 sample (Figure 4b). The effect of GTR on hardness, with an average of 91.1 ± 0.6 Shore A (Figure 4c), and density, with an average of 1.22 ± 0.01 g/cm^3^ (Figure 4d), was negligible.

Recently, Zhao et al. [45] developed and comprehensively characterized 3D-printed TPU/carbon black composites with carbon black content in the range of 1–7 wt%, which were characterized by a tensile strength in the range of 6.8–7.1 MPa and an elongation at break in the range of 193–892%. The tensile properties (especially the elongation at break) of studied materials decreased with increasing carbon black content.

Álvarez-García and Martín-Martínez [46] showed that a higher amount of carbon black in TPU/carbon black composites resulted in the formulation of agglomerates due to poor wettability between the non-polar carbon particles and the polar polyurethane matrix. This phenomenon resulted in the deterioration of the mechanical properties of TPU/carbon black composites with higher carbon black content.

Toncheva et al. [47] studied the effect of GTR content in the range of 0–30 wt% on thermoplastic polyurethane (TPU) powder/GTR composites prepared through 3D printing using selective laser sintering. It was found that a higher content of GTR resulted in the deterioration of mechanical properties and, at the same time, a higher porosity of TPU/GTR composites. The negative effect of higher porosity in the studied composites can be partially reduced by the proper adjustment of 3D printing conditions. Tensile strength and elongation at break of the obtained composites were in the range of 1.2–8.1 MPa and 55–148%, respectively.

Kyriakidis et al. [48] investigated the static and dynamic mechanical properties of TPU/GTR composites prepared through selective laser sintering. The results showed that using GTR up to 20 wt% improves the energy absorption and damping performance of TPU/GTR composites due to the cross-linked structure of GTR. Prepared TPU/GTR composites were characterized by a tensile strength in the range of 11.9–12.7 MPa and an elongation at break in the range of 415–499%.

Kohári and Bárány [49] developed thermoplastic elastomers wherein the TPU matrix was substituted by GTR or devulcanized GTR in the range of 0–50 wt%. It was observed that, above a 10 wt% GTR content, the mechanical properties of the TPU/GTR composites were reduced. The tensile strength and elongation at break of TPU/GTR composites were in the range of 5.4–29.1 MPa and 304–714%, respectively. In the conclusion, the authors highlighted that TPU/GTR composites can be used as filaments for fused deposition modeling to make rubber-like (color, smell and feel) products.

The above presented works indicated that the obtained TPU/CB/GTR composites, with a tensile strength in the range of 5.9–8.5 MPa, elongation at break in the range of 23–45%, and density of approximately 1.2 g/cm^3^, have a huge potential for application in additive manufacturing technologies, which will be the aim of our further studies.

### 3.5. Surface Analysis and Morphology

**Figure 5:**
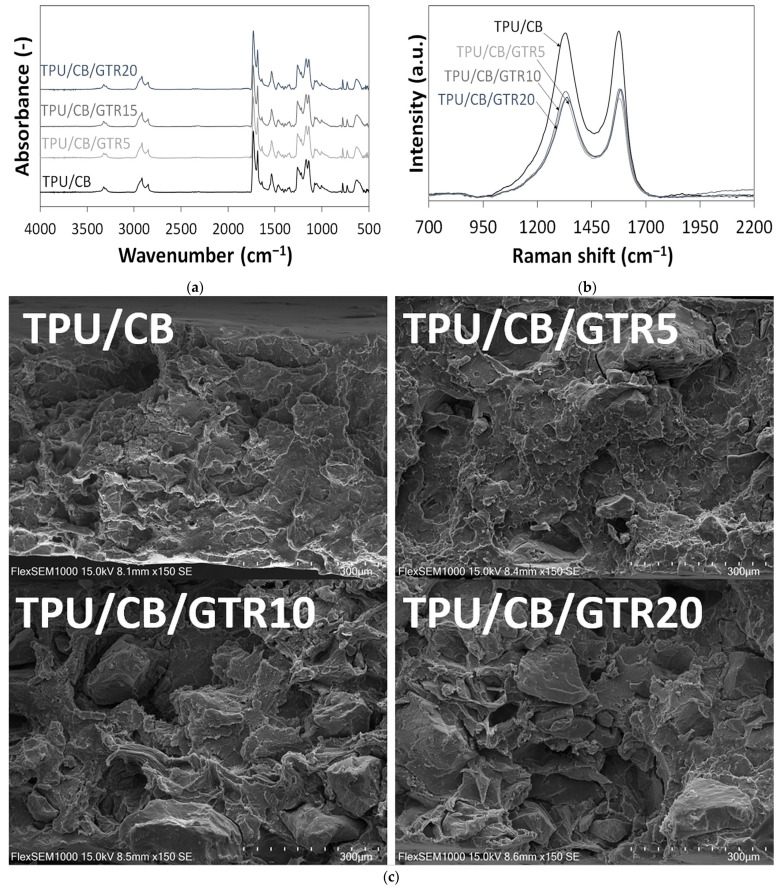
Surface analysis and morphology of studied composites: (**a**) FTIR; (**b**) Raman and (**c**) SEM images (magnification ×150).

The effect of GTR content on the chemical structure of TPU/CB/GTR composites was investigated using FTIR spectroscopy, and the results are presented in Figure 5a.

As can be observed, all FTIR spectra are very similar and characteristic of a polyurethane matrix. The bands at 3290–3315 cm^−1^ are related to the N–H stretching vibration, while the bands at 1531 cm^−1^ and 1161 cm^−1^ are attributed to the N–H bending and in plane movement, respectively [46]. A strong signal at 1683–1728 cm^−1^ corresponds to C=O stretching, while the band at 1251 cm^−1^ is related to C–N and C–O group stretching. FTIR analysis indicates that the effect of GTR on the chemical structure of TPU/CB/GTR composites was negligible and confirms only physical interactions between mixed components.

Raman spectroscopy was used to characterize TPU/CB and TPU/CB/GTR composites and to assess the effect of the GTR content. The obtained results are presented in Figure 5b. All measured spectra exhibited the D band (~1332 cm^−1^) and G band (~1580 cm^−1^), which are characteristic of carbon-based composites. These bands originate from conductive carbon black nanofiller added to the TPU matrix and also carbon black present in GTR [50]. As can be observed, the TPU/CB/GTR composites signals from the D band and G band become less intensive compared to the TPU/CB composite as a reference. However, it should be highlighted that the effect of the GTR content on the ratio between the D band intensity (I_D_) and G band intensity (I_G_) was negligible. The I_D_/I_G_ ratio calculated for the TPU/CB composite was 0.99. For TPU/CB/GTR composites, the I_D_/I_G_ ratio was in the range of 0.93–0.99 and a slightly decreased I_D_/I_G_ parameter was observed for the TPU/CB/GTR20 sample, which corresponds with a higher electrical conductivity (see Figure 2). The low I_D_/I_G_ ratio (around 1.0) indicates the presence of carbon black on the surface of the studied materials [51].

Scanning electron microscopy (SEM) was used to investigate the breaking mechanism of TPU/CB/GTR composites. SEM images of surfaces created by breaking the samples in the tensile test at a speed of 500 mm/min are shown in Figure 5c. As can be observed, the addition of GTR to the TPU/CB matrix resulted in phase separation and void formation, which increased with a higher GTR content in the TPU/CB/GTR composites. This is due to the weak compatibility between the cross-linked GTR and TPU matrix and crack propagation in the interphase during uniaxial stretching, affecting the deterioration of the mechanical properties of TPU/CB/GTR composites (see Figure 4a,b).

### 3.6. Electrochemical Properties

**Figure 6:**
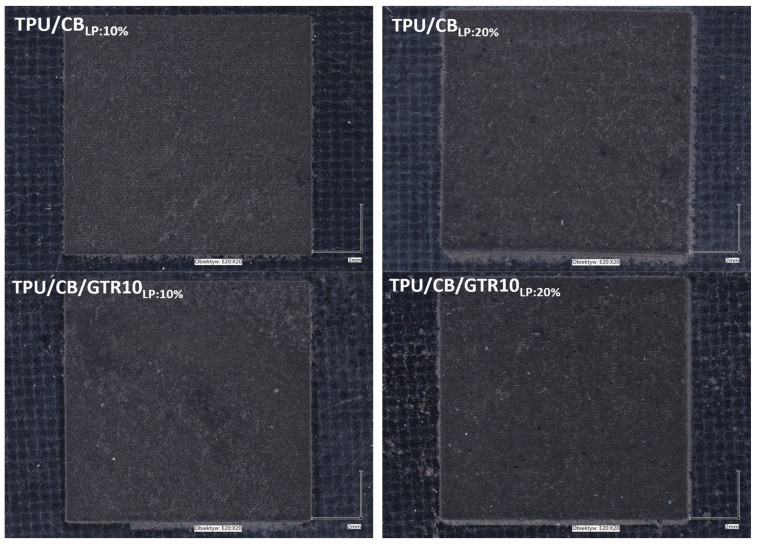
The appearance of sample surfaces after laser treatment (magnification ×20).

Considering cost savings, rheological behavior, and mechanical and electrical properties, the TPU/CB/GTR10 sample was selected for the further investigation of electrochemical activity. Prior to electrochemical measurements, sample surface activation was performed using a laser with a maximum power of 40 W, and, based on the preliminary studies, 10% and 20% of the maximum laser power (LP) were chosen for investigation. The appearance of laser-treated samples is presented in Figure 6.

**Figure 7:**
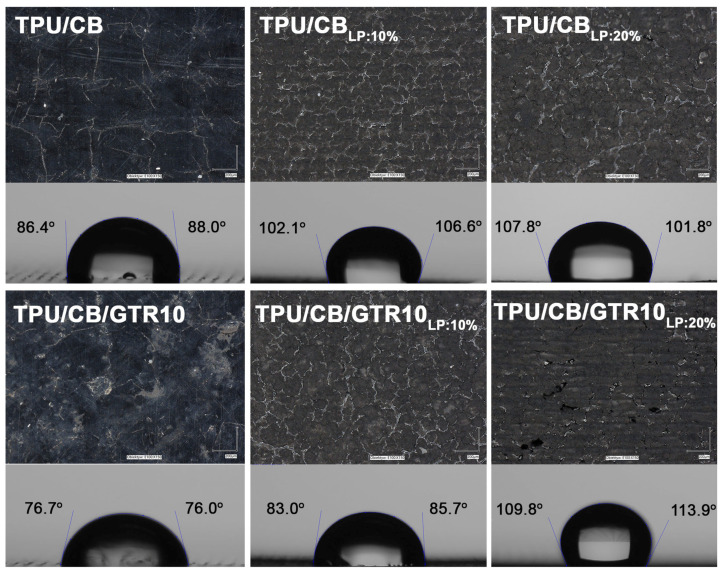
Contact angle measurements and surface morphology (magnification ×150) before and after laser treatment for samples TPU/CB and TPU/CB/GTR10.

Contact angle and digital microscopy were used to assess the changes in the sample surface before and after laser treatment. The obtained results are presented in Figure 7. As can be observed, the higher power of laser activation of the studied materials significantly increased the contact angle and caused the surfaces of the studied samples to be less developed. This indicates a melting of the TPU matrix during laser treatment and a higher hydrophobicity of the sample surface. The application of 10 wt% micronized GTR resulted in a lower contact angle compared to the TPU/CB sample. This can be explained by the presence of voids and roughness in the TPU/CB/GTR10, which corresponds with the observation made through SEM (see Figure 5c). Furthermore, it was noticed that 20% of the laser power caused a significant increase in the contact angle measured for the TPU/CB/GTR10 sample, for which also the smoothest surface was observed. This is due to a higher content of carbon black in TPU/CB/GTR10 compared to TPU/CB (see Figure 3), which might improve heat transfer in the material after laser treatment.

**Figure 8:**
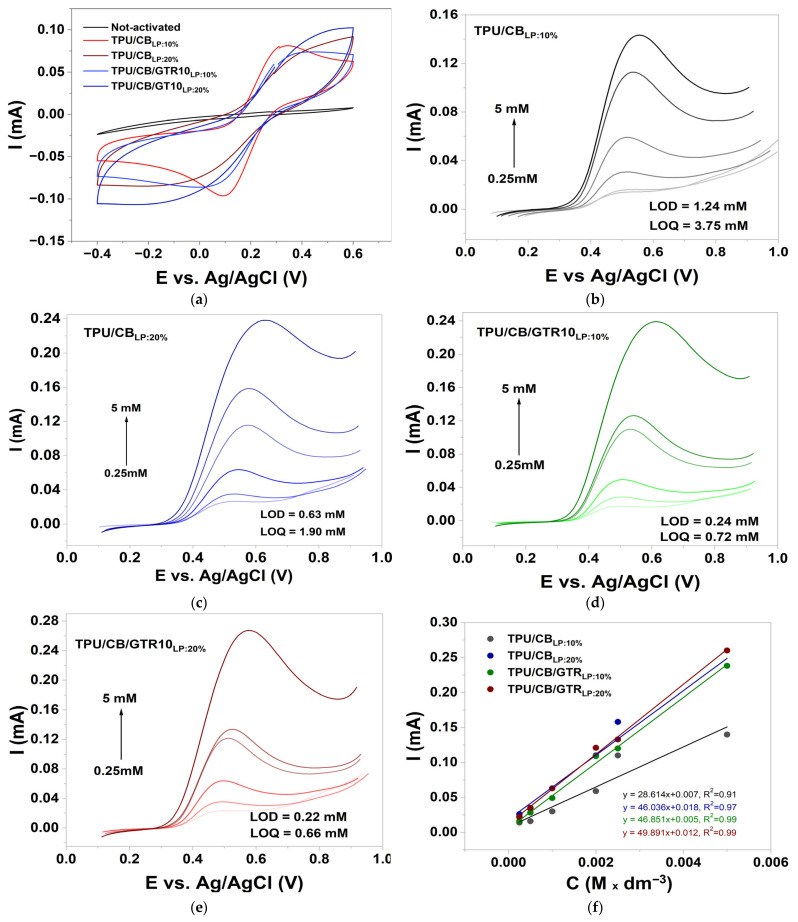
Electrochemical studies for TPU/CB and TPU/CB/GTR10 composites: (**a**) CV scans of 2.5 mM [Fe(CN)_6_]^3−/4−^ in 0.01 M PBS for not-activated and activated composites; (**b**–**f**) DPV scans for paracetamol detection in 0.01 M PBS and corresponding calibration curves.

Electrochemical measurements using the ferri-/ferrocyanide redox probe are presented in Figure 8a. The results revealed clear differences between pristine and laser-activated composite surfaces.

The cyclic voltammetry (CV) curves of non-activated electrodes show a strongly flattened shape hysteresis loop and the absence of characteristic anodic and cathodic peaks, indicating that the electroactivity of the composite is limited and the CB filler is covered by the polymer matrix. After laser activation at a laser power (LP) of both 10% and 20%, the current response increases significantly and the CV loops widen, indicating an increased number of available conductive paths and an expanded active electrode surface.

It is worth noting that laser activation simultaneously increases the surface hydrophobicity, as confirmed by the contact angle measurements. Despite the lower wettability, which theoretically could impede electrode–electrolyte contact, the observed electrochemical signal is significantly stronger. This indicates that changes in the microstructure and conductive network, rather than the wetted surface properties, have a decisive impact on the conductivity. The laser causes local melting and microablation of the TPU matrix, exposing and interconnecting the CB domains and GTR in the composite, resulting in a significant improvement in electron transport.

Electrochemical sensors enable the rapid detection of this analyte in various body fluids without lengthy sample preparation. To test the potential application of the studied composites as electrochemical sensors, we chose paracetamol as the analyte, as it is one of the most commonly used analgesics and antipyretics worldwide. Furthermore, paracetamol undergoes a reversible redox reaction, allowing for sensitive and selective detection even at low concentrations.

During the studies, differential pulse voltammetry (DPV) was used, and the results are presented in Figure 8b–f. It was found that the pulse peaks for the 20% laser-activated samples were higher compared to those activated at 10% laser power, indicating a relatively enhanced charge-transfer efficiency. These results together indicate that laser activation changes an initially polymer-rich surface into a carbon-dominated, electroactive interface that allows efficient redox reactions in both TPU/CB and TPU/CB/GTR composites.

The results in Figure 8f showed that the use of the TPU/CB/GTR10_LP:20%_ composite achieved the lowest LOD for paracetamol equal to 0.22 mM (LOQ = 0.66 mM), a value approximately three times lower than that recorded for the TPU/CB_LP:20%_ composite.

Compared with the information reported in the literature, the LOD of paracetamol using prepared composites is very high. However, considering the Rumack–Matthew nomogram used for paracetamol poisoning and toxicity assessment [52], TPU/CB/GTR10_LP:20%_ might be tested as a material for paracetamol overdose diagnosis. Usually, a plasma paracetamol concentration above 0.662 mM (100 μg/mL) at 4 h post-ingestion indicates the necessity of treatment to prevent hepatotoxicity and liver failure [53].

The high LOD for paracetamol in flexible and conductive TPU/CB/GTR composites is probably correlated with the relatively low content of conductive carbon black (15 wt%) in the studied materials. For example, Kozłowska et al. [54] showed that the use of a commercial composite material, a polylactic acid/carbon black composite (tradename: Proto-Pasta) with a conductive carbon black content of approximately 21.4wt%, allowed them to achieve detection limits of 1.31 μM. Recently, Oliveira et al. [55] developed a thermoplastic polyurethane/carbon black composite with a 35–45 wt% conductive carbon black content and achieved an LOD for paracetamol of 1.74 μM.

As presented above, the polymer matrix type combined with the content of conductive carbon black (and also carbon black specification, e.g., surface area, particle size, etc.) seems to be crucial for the detection of redox compounds. Therefore, further studies in this field are necessary to improve the limit of detection for sensors based on TPU/CB composites with GTR as a low-cost modifier.

## 4. Conclusions

In this study, flexible and conductive polymer composites from thermoplastic polyurethane powder filled with carbon black and micronized ground tire rubber were developed. The rheological, mechanical, thermal, morphological and electrochemical behavior of thermoplastic polyurethane/carbon black were investigated as a function of the micronized ground tire rubber content (in the range of: 5–20 wt%).

It was observed that a higher GTR content resulted in a decreased melt mass–flow rate for the studied composites. This is due to the limited flowability of the composite material from the application of cross-linked rubber particles. However, it should be pointed out that, for TPU/CB composites with 5 wt%, the melt mass–flow rate was higher than in the case of the TPU/CB sample, which indicates the main chain scission and cross-linking degradation of GTR during high-shear mixing.

The mechanical properties and thermal stability of the studied composites decreased with an increasing GTR content. This is due to the poor compatibility and interfacial adhesion between GTR and the TPU matrix, confirmed by scanning electron microscopy observations. FTIR and Raman spectroscopy showed that the effect of GTR on the chemical structure of TPU/CB/GTR composites was negligible, which confirms the physical interactions between GTR and the TPU matrix.

It was found that the addition of micronized GTR improves the electrical properties of TPU/CB/GTR composites. This is due to two factors: (i) a higher content of carbon black in the system with a higher amount of GTR and (ii) the low compatibility of cross-linked GTR and the polyurethane matrix. This resulted in phase separation, which promotes local carbon-rich domains and facilitates conductive pathway formation.

The most developed redox response was observed for the composite containing GTR, especially activated at 20% laser power. Synergy between a higher laser absorption by GTR particles and microstructural rearrangement leads to the creation of the most effective conducting paths, reflected in the highest anodic and cathodic current values. In summary, the electrochemical activity increase after laser activation is mainly due to the creation of a continuous and well-connected conduction network—not to any improvement in the surface wettability, which actually deteriorates.

The application of micronized ground tire rubber as a substitute of thermoplastic polyurethane powder in the range of 5–20 wt% reduces the manufacturing cost in the range of 5.6–22.6%.

This research work confirms that the further development of soft electronics using ground tire rubber as a low-cost and valuable source of raw materials is fully justified. Considering the obtained results, further investigations in this field should focus on: (i) the optimization of the composition and processing conditions of thermoplastics/GTR systems to achieve a good correlation between the price and performance properties of materials; (ii) compatibilization strategies, including GTR devulcanization and reactive blending (e.g., reactive extrusion, GTR grafting or encapsulation, dynamic vulcanization, etc.), focused on developing of GTR-based composites; (iii) problems related to recyclability and volatile organic compound emission during the processing of developed materials to assess their impact on human health and the environment; (iv) the application of thermoplastic/GTR systems in additive manufacturing technologies; (v) the development of surface treatment methods and the standardization of protocols related to electrochemical analysis to allow for an easier comparison of obtained results with other research groups.

## Figures and Tables

**Table 1:** Composition and coding of TPU/CB/GTR composites.

Component	Sample Coding				
	TPU	TPU/CB	TPU/CB/GTR5	TPU/CB/GTR10	TPU/CB/GTR20
TPU	100	85	80	75	65
CB		15	15	15	15
GTR			5	10	20

**Table 2:** Thermal decomposition temperatures and char residue of studied materials.

Sample Code	Decomposition Temperature (°C)				Char Residue (wt%)
	T_−2%_	T_−5%_	T_−10%_	T_−50%_	
TPU	297	312	327	381	2.2
TPU/CB	285	304	321	398	16.0
TPU/CB/GTR5	290	308	325	392	20.3
TPU/CB/GTR10	286	306	324	386	22.0
TPU/CB/GTR20	279	299	316	361	25.6

## Data Availability

The original contributions presented in this study are included in the article. Further inquiries can be directed to the corresponding author.

## Abbreviations

The following abbreviations are used in this manuscript:

CB	Carbon black
CV	Cyclic voltammetry
DTG	Derivative thermogravimetry
DSC	Differential scanning calorimetry
DPV	Differential pulse voltammetry
FTIR	Fourier transform infrared spectroscopy
GTR	Ground tire rubber
LOD	Limit of detection
LOQ	Limit of quantification
LP	Laser power
MFR	Melt mass–flow rate
MVR	Melt volume–flow rate
SEM	Scanning electron microscopy
TGA	Thermogravimetric analysis
TPE	Thermoplastic elastomer
TPU	Thermoplastic polyurethane
